# Disturbed Expression of Splicing Factors in Renal Cancer Affects Alternative Splicing of Apoptosis Regulators, Oncogenes, and Tumor Suppressors

**DOI:** 10.1371/journal.pone.0013690

**Published:** 2010-10-27

**Authors:** Agnieszka Piekielko-Witkowska, Hanna Wiszomirska, Anna Wojcicka, Piotr Poplawski, Joanna Boguslawska, Zbigniew Tanski, Alicja Nauman

**Affiliations:** 1 Department of Biochemistry and Molecular Biology, The Medical Centre of Postgraduate Education, Warsaw, Poland; 2 Regional Hospital, Ostroleka, Poland; Centre de Regulació Genòmica, Spain

## Abstract

**Background:**

Clear cell renal cell carcinoma (ccRCC) is the most common type of renal cancer. One of the processes disturbed in this cancer type is alternative splicing, although phenomena underlying these disturbances remain unknown. Alternative splicing consists of selective removal of introns and joining of residual exons of the primary transcript, to produce mRNA molecules of different sequence. Splicing aberrations may lead to tumoral transformation due to synthesis of impaired splice variants with oncogenic potential. In this paper we hypothesized that disturbed alternative splicing in ccRCC may result from improper expression of splicing factors, mediators of splicing reactions.

**Methodology/Principal Findings:**

Using real-time PCR and Western-blot analysis we analyzed expression of seven splicing factors belonging to SR proteins family (SF2/ASF, SC35, SRp20, SRp75, SRp40, SRp55 and 9G8), and one non-SR factor, hnRNP A1 (heterogeneous nuclear ribonucleoprotein A1) in 38 pairs of tumor-control ccRCC samples. Moreover, we analyzed splicing patterns of five genes involved in carcinogenesis and partially regulated by analyzed splicing factors: RON, CEACAM1, Rac1, Caspase-9, and GLI1.

**Conclusions/Significance:**

We found that the mRNA expression of splicing factors was disturbed in tumors when compared to paired controls, similarly as levels of SF2/ASF and hnRNP A1 proteins. The correlation coefficients between expression levels of specific splicing factors were increased in tumor samples. Moreover, alternative splicing of five analyzed genes was also disturbed in ccRCC samples and splicing pattern of two of them, Caspase-9 and CEACAM1 correlated with expression of SF2/ASF in tumors. We conclude that disturbed expression of splicing factors in ccRCC may possibly lead to impaired alternative splicing of genes regulating tumor growth and this way contribute to the process of carcinogenesis.

## Introduction

Renal cell carcinoma (RCC) is the most common solid lesion of the kidney and represents ∼3% of all human malignancies. Each year in Europe about 40 000 new cases of RCC are diagnosed and approximately 20 000 patients die of the disease [1]. The vast majority (80%) of RCC cases are histologically classified as clear cell renal cell carcinomas (ccRCC), originating from proximal tubules of the kidney. The molecular basis of ccRCC is not fully understood. Although several molecular markers have been proposed, neither of them has been approved for routine clinical use [2].

One of the cellular processes, often disturbed in cancers, is alternative splicing, the process of selective removal of introns and joining of residual exons, in which mRNA molecules of various sequences are produced. Aberrant alternative splicing may lead to tumoral transformation [3]. Impaired alternative splicing of several genes was also reported in ccRCC. For instance, in our previous work we found ccRCC-specific imbalanced expression of type 1 iodothyroine deiodinase (DIO1) splicing variants [4], [5] and untranslated regions of thyroid hormone receptor TRβ1 [6]. Several other reports showing ccRCC-specific disturbances of alternative splicing include alterations in mRNA processing of Mcl-1 [7], TCF-4 [8], survivin [9], and OGG1 [10]. Abnormally spliced variants of genes described above are rarely effects of mutations in genes coding for spliced transcripts and the sources of disturbances in alternative splicing are usually unknown.

Alternative splicing is a complicated process, involving a significant number of proteins including splicing factors called serine-arginine rich proteins (SR proteins) [11]. The family of SR proteins consists of at least twenty members of which seven: SF2/ASF (encoded by gene: SFRS1), SC35 (gene: SFRS2), SRp20 (gene: SFRS3), SRp75 (gene: SFRS4), SRp40 (gene: SFRS5), SRp55 (SFRS6), and 9G8 (SFRS7) constitute the group of “classical” SR proteins. These factors bind to sequences called splicing enhancers, located in exons (ESEs, exonic splicing enhancers) or in introns (ISEs, intronic splicing enhancers). Binding of SR proteins to splicing enhancers promotes exon inclusion. The splicing reaction is also regulated by a large number of non-SR factors, such as hnRNPs (heterogeneous nuclear ribonucleoproteins) which mainly bind to sequences of splicing silencers and act as splicing repressors [12]. Thus, the final result of alternative splicing is an effect of the concert action of antagonistically acting splicing factors. One pair of splicing factors exhibiting opposite activities is SF2/ASF (an SR protein) and hnRNP A1 (heterogeneous nuclear ribonucleoprotein A1; a non-SR protein) [13]. Excess of SF2/ASF promotes proximal 5′ splice site selection while of hnRNP A1 favours distal 5′ splice site. Specific members of SR family may also act antagonistically (e.g. SF2/ASF and SRp20 [14], or SF2/ASF and SC35 [15]). Thus, relative levels of specific splicing factors contribute to regulation of alternative splicing, specific for tissue type and developmental stage.

It is known that disturbances in alternative splicing may contribute to carcinogenesis due to production of tumor-suppressive or oncogenic variants of gene transcripts, affecting proliferation, cell motility, and apoptosis susceptibility [16]. Improperly spliced transcript variants may also serve as tumor biomarkers [17]. The growing body of evidence suggests that splicing factors may be directly involved in the process of carcinogenesis, acting as proto-oncogenes [18] or regulating splicing and activity of proto-oncogenes [19], tumor suppressors [18] and apoptosis regulators [20]. Disturbed expression of splicing factors was reported in several types of cancers [11]. In our recent paper we showed that the expression of two splicing factors, SF2/ASF and hnRNP A1 is disturbed in ccRCC [4] but to our knowledge the expression of splicing factors as a group had never been analyzed in ccRCC.

In this paper we hypothesized that the observed disturbances of alternative splicing in ccRCC may be a consequence of changes in expression of splicing factors, in particular, of aberrations of quantitative relations between them. To address this problem, we analyzed expression of seven classical splicing factors: SF2/ASF, SC35, SRp20, SRp75, SRp40, SRp55, 9G8 and a non-SR factor, hnRNP A1. In addition, to investigate the consequences of disturbed expression of splicing factors, we analyzed existence and expression of transcripts of five genes involved in tumorigenesis, that are known to be alternatively spliced and partially regulated by the analyzed splicing factors. We found that the expression of splicing factors as well as alternative splicing of analyzed genes were disturbed in majority of analyzed tissue samples. We conclude that disturbed expression of splicing factors in ccRCC may possibly lead to impaired alternative splicing of genes regulating tumor growth and therefore contributing to the process of carcinogenesis.

## Materials and Methods

### Tissue specimens

Tissue samples were obtained from unilateral nephrectomies performed on patients with clear cell renal cell cancer (38 patients) with permission of the Ethical Committee of Human Studies (The Medical Centre of Postgraduate Education). Samples were divided into two groups: cancer tissues (n = 38, T) and control tissues (paired normal tissue from the opposite pole of the malignant kidney with no histological evidence of tumor; n = 38, C). Clear cell renal cell cancer was diagnosed by histology according to WHO criteria [21]. Tumors were divided into three groups, depending on the grade of differentiation: G1 (well differentiated), G2 (intermediate grade of differentiation), G3 (poorly differentiated cancers).

### RNA isolation

Total cellular RNA was isolated from ∼100 mg of frozen tissue using GeneMATRIX Universal RNA Purification Kit (EURx, Gdansk, Poland), according to the manufacturer's protocol.

### Reverse transcription

600 ng of total RNA was reverse transcribed using RevertAidTM H Minus First Strand cDNA Synthesis Kit (Fermentas, Vilnius, Lithuania) and oligo-dT primers according to the manufacturer's protocol. For subsequent PCR analysis 1 ul of cDNA was used. For real time PCR reactions 1 ul of 5x diluted cDNA was taken.

### PCR analysis of alternative splicing

PCR reaction was performed on 1 µl of 5x diluted cDNA using Perpetual OptiTaq DNA Polymerase HOT START (EURx, Gdansk, Poland) under conditions 95°C, 10 min. (initial denaturation), followed by 35 cycles: [95°C, 30 s; 58°C, 30 s; 72°C, 30 s], final elongation: 61°C, 10 min. Sequences of specific primers (Table S1) were taken from the previously published reports for: RON [19], Caspase-9 [22], CEACAM-1 [23], Rac1 [24], and GLI1 [25]. The PCR products were electrophoresed in 1–2% agarose gel stained with ethidium bromide.

### Real-time PCR

Semi-quantitative real-time PCR was performed using LightCycler® 480 DNA SYBR Green I Master (Roche Diagnostics, Mannheim, Germany) in triplicate according to manufacturer's protocol. Sequences of the primers are shown in Table S2. Conditions for real-time PCR were as follows: initial denaturation: 95°C, 10 min., 45 cycles: (95°C, 15 s, 57°C, 15 s; 72°C, 15 s); followed by melting curve analysis: (95°C, 5 min; 65°C, 1 min; continuous reading of fluorescence from 65°C to 97°C with 0.11°C/s ramp rate and 5 acquisitions per each °C). Results were normalized to expression of 18sRNA host-gene *RN18S1*. The stability of expression of 18sRNA was validated and confirmed in initial pre-analysis of 32 pairs of control and tumor samples by comparison with ACTB expression (Figure S1). The ACTB primers were published elsewhere [6].

### Protein extraction and Western blot analysis

For Western analysis, twelve representative pairs of tumor and control samples were taken. Tissue samples were homogenized in a buffer containing 150 mM NaCl, 1% Triton X-100, 50 mM Tris-HCl pH 8.0, protease inhibitor cocktail (Sigma-Aldrich, Saint Louis, MO), and 0.5 mM PMSF. The homogenate was incubated with shaking for 2 hours at 4°C and centrifuged at 12,000 rpm for 20 min, at 4°C. The obtained supernatant was used for protein concentration analysis with Thermo Scientific Pierce BCA Protein Assay (Pierce Biotechnology, Rockford, IL) according to standard protocol. The protein extracts were divided into 30 µl aliquots and stored at −70°C.

For SF2/ASF Western blotting, 30 µg of protein extract was resolved by 10% SDS-PAGE. After electrophoresis the proteins were transferred onto nitrocellulose membranes that were subsequently blocked overnight at 8°C in 5% non-fat milk in TBS-T buffer (10 mM Tris-HCl, 150 mM NaCl, 0.1% Tween-20; pH 7.6). The membranes were washed three times in TBS-T for 10 min at RT, and incubated overnight at 8°C with anti-SF2/ASF antibody (cat. no.: 32–4500, Invitrogen, Carlsbad, Ca) diluted 1: 500 in TBS-T buffer with 5% non-fat milk. After washing 3 times for 10 min with TBS-T, the membranes were incubated for 1 h at RT with horseradish peroxidase-conjugated goat anti-mouse secondary antibody (1∶10000, DakoCytomation, Glostrup, Denmark), and washed 3 times for 10 min with TBS-T.

Western blotting of hnRNP A1 was performed as for SF2/ASF analysis using 15 µg of protein extract and anti-hnRNP A1 antibody (cat. no.: ab10685, Abcam plc, Cambridge, UK).

Proteins were detected by an enhanced-chemiluminescence detection system (Supersignal West Pico Chemiluminescent Substrate, Pierce Biotechnology, Rockford, IL) according to standard procedures. Subsequently, the membranes were stripped, blocked and incubated with anti-β-actin antibody (cat. no. ab6276, Abcam plc, Cambridge, UK) diluted 1∶10000 in TBS-T buffer for 1 h at RT, washed three times in TBS-T buffer and further processed as described for SF2/ASF procedure.

The amount of specific protein was estimated densitometrically after normalization to expression of β-actin.

### Prediction of splicing factor binding motifs

Analysis of CEACAM1 exon 7 sequence was performed with ESE finder software [26]. For the prediction of SF2/ASF binding sites, two matrices were used: “SF2/ASF/IgM-BRCA1” and “SF2/ASF”. These two matrices were derived in different context (different minigenes and size of random sequence libraries in SELEX [27]). The thresholds used for prediction were: 1.956 (SF2/ASF), 1.867 (SF2/ASF/IgM-BRCA1), 2.383 (SC35), 2.670 (SRP40), and 2.676 (SRp55). The sequence of exon 7 was derived from CEACAM1 transcript variant 1 (Acc. no. NM_001712.3).

### Statistical analysis

The Shapiro-Wilk test was used to determine normality of data distribution. Normally distributed data were analyzed by paired t-test and non-parametric data by Wilcoxon matched pairs test. *p<*0.05 was considered statistically significant. Visualization of correlation matrix was done using corrplot on R platform [28].

## Results

### The mRNA expression of splicing factors is disturbed in at least half of ccRCC samples

Splicing factors belonging to the group of SR proteins comprise a large number of structurally and functionally related proteins [11]. In our study we focused on the group of “classical” SR proteins, i.e. SF2/ASF (encoded by gene: SFRS1), SC35 (SFRS2), Sp20 (SFRS3), SRp75 (SFRS4), SRp40 (SFRS5), SRp55 (SFRS6), and 9G8 (SFRS7). We have also analyzed expression of a non-SR splicing factor, hnRNP A1 that is generally believed to act as antagonist of SR proteins.

**Figure 1:**
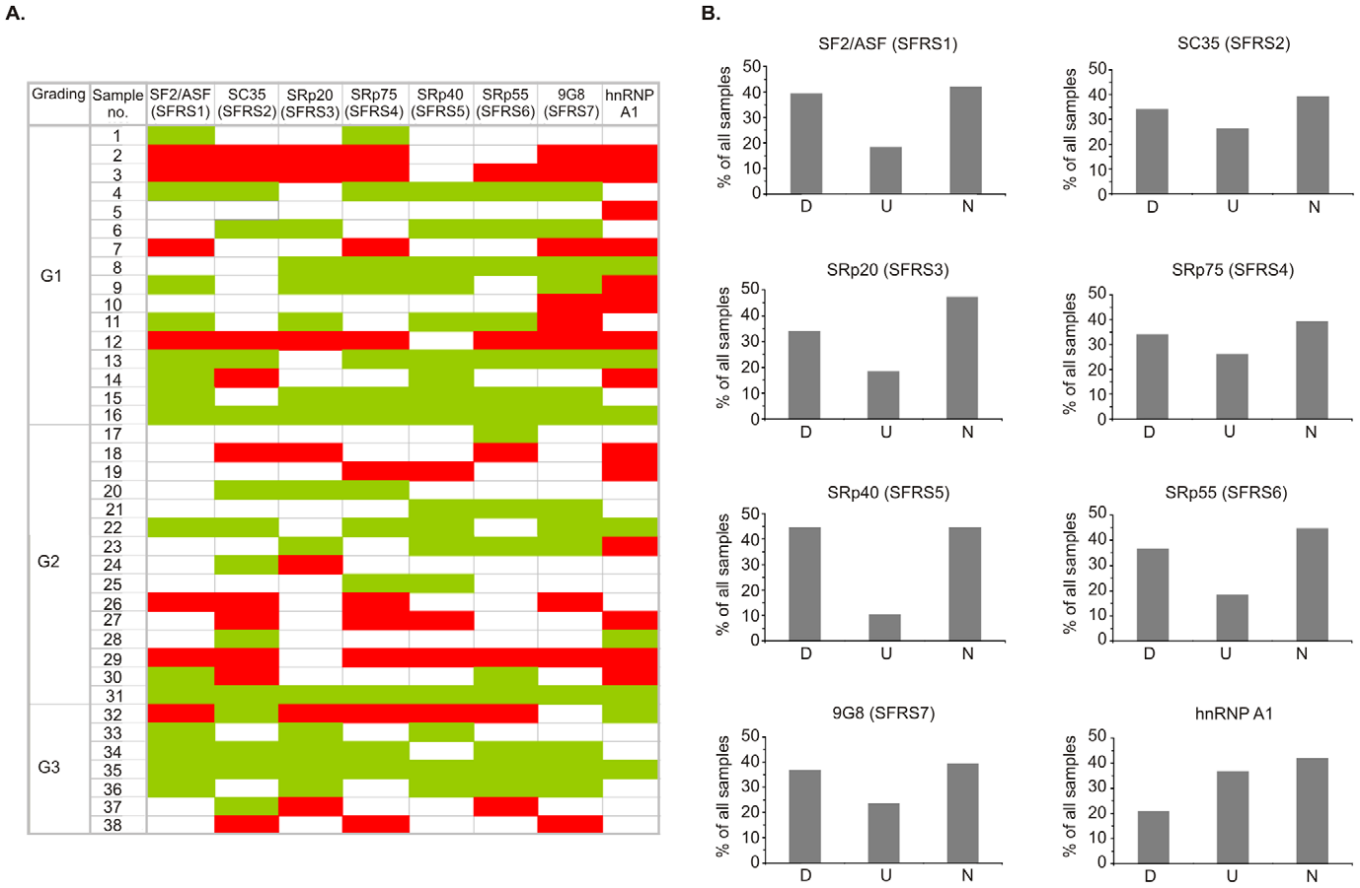
Expression of splicing factors mRNA in ccRCC. **A.** Changes in expression of particular pairs of control and tumor samples. The diagram was performed based on the data from real-time PCR analysis (Figure S2). Colors represent expression ratio between control and tumor samples. Green: downregulation in tumor samples. Red: upregulation in tumor samples. White: no difference in expression levels. Tumor grading (G1, G2, G3) is shown. **B.** Distribution of changes in mRNA expression of splicing factors. D: group of samples with tumor-specific downregulation of expression; U: group of samples with tumor-specific upregulation of expression; N: group of samples that did not differ in expression between control and tumor samples. The threshold of 30% difference in expression between control and tumor samples was used to classify samples.

Using real-time PCR we found that the pattern of expression of splicing factors differed between individual patients, as well as between control and tumor samples of a particular patient. mRNA expression of all analyzed splicing factors was disturbed in about 50–60% (depending on the splicing factor analyzed) of tumor samples compared with paired normal tissues (Fig. 1). These changes of expression were due to down- or upregulation of the analyzed genes and allowed us to divide all tumor samples into three pools: D, U, and N in which the expression of genes was downregulated, upregulated or not changed, respectively. The direction of changes did not correlate with tumor grade of differentiation (Fig. 1A).

**Figure 2:**
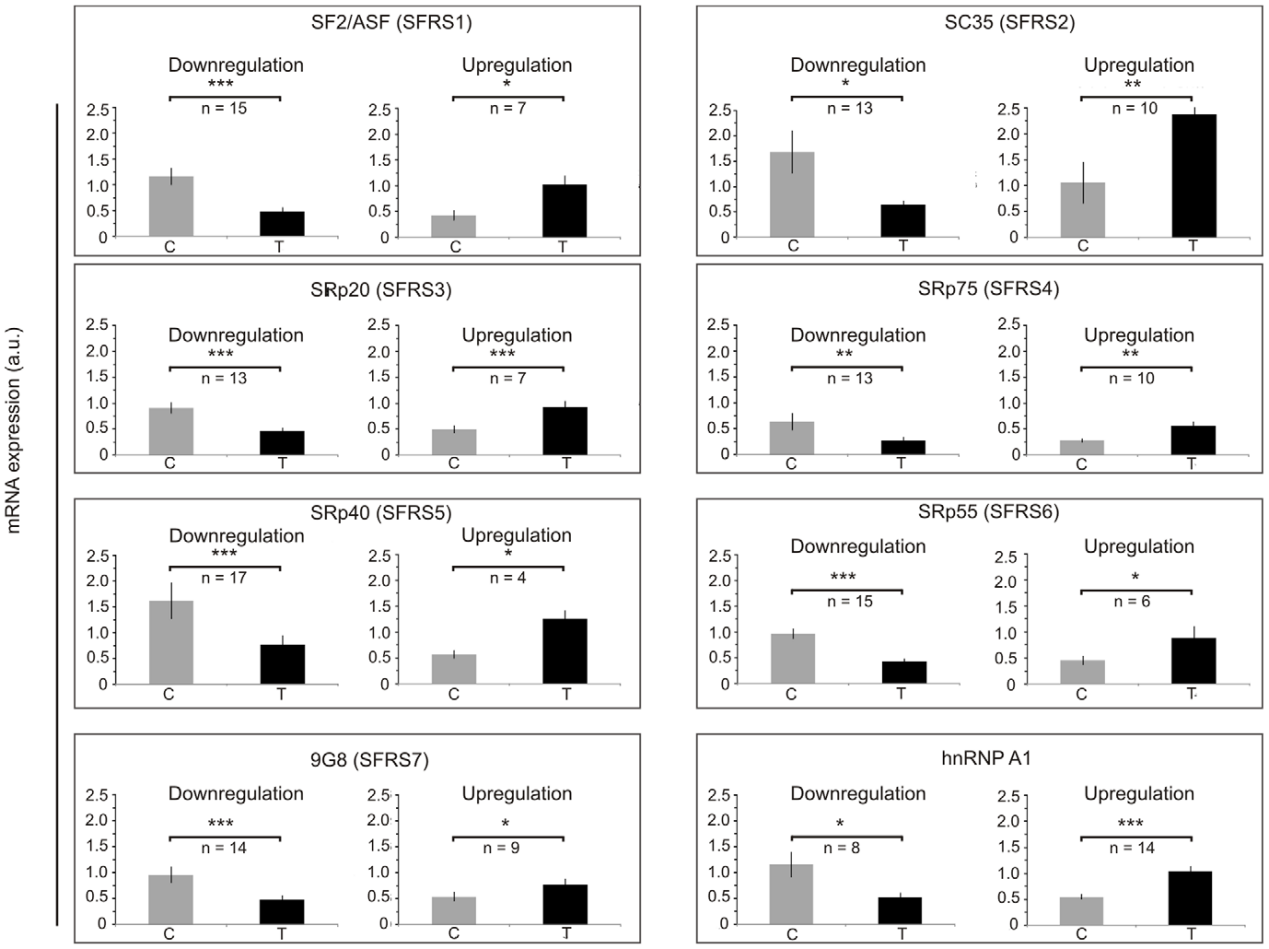
Disturbances of expression of splicing factors mRNA. Expression of each splicing factor is shown in two groups of samples: with tumor-specific downregulation (D) (left) and upregulation (U) (right). The threshold of 30% difference in expression between control and tumor samples was used to classify samples. C: control samples, T: tumor samples. The data are given as mean ± S.E.. Statistical analysis was performed using paired t-test. * p<0.05; ** p<0.01; *** p<0.001.

The number of samples in each pool varied from n = 8 for D and n = 14 for U (for hnRNP A1) to n = 17 for D and n = 4 for U (for SRp40) (Fig. 2). For majority of samples with altered expression, pool D was the most abundant (34–45% of all analyzed samples). The only exception was expression of hnRNP A1 that was downregulated in only 21% of samples and upregulated in 37% of all analyzed samples. Downregulation of genes in pools D varied from 1.9 fold (SRp20) to 2.6 fold (SC35 ad SRp75) when compared with control samples. Genes in pools U were upregulated from 1.4 fold (9G8) to 2.4 fold (SF2/ASF) when compared with control samples.

### Quantitative relations between splicing factors differ between tumor and control samples

**Figure 3:**
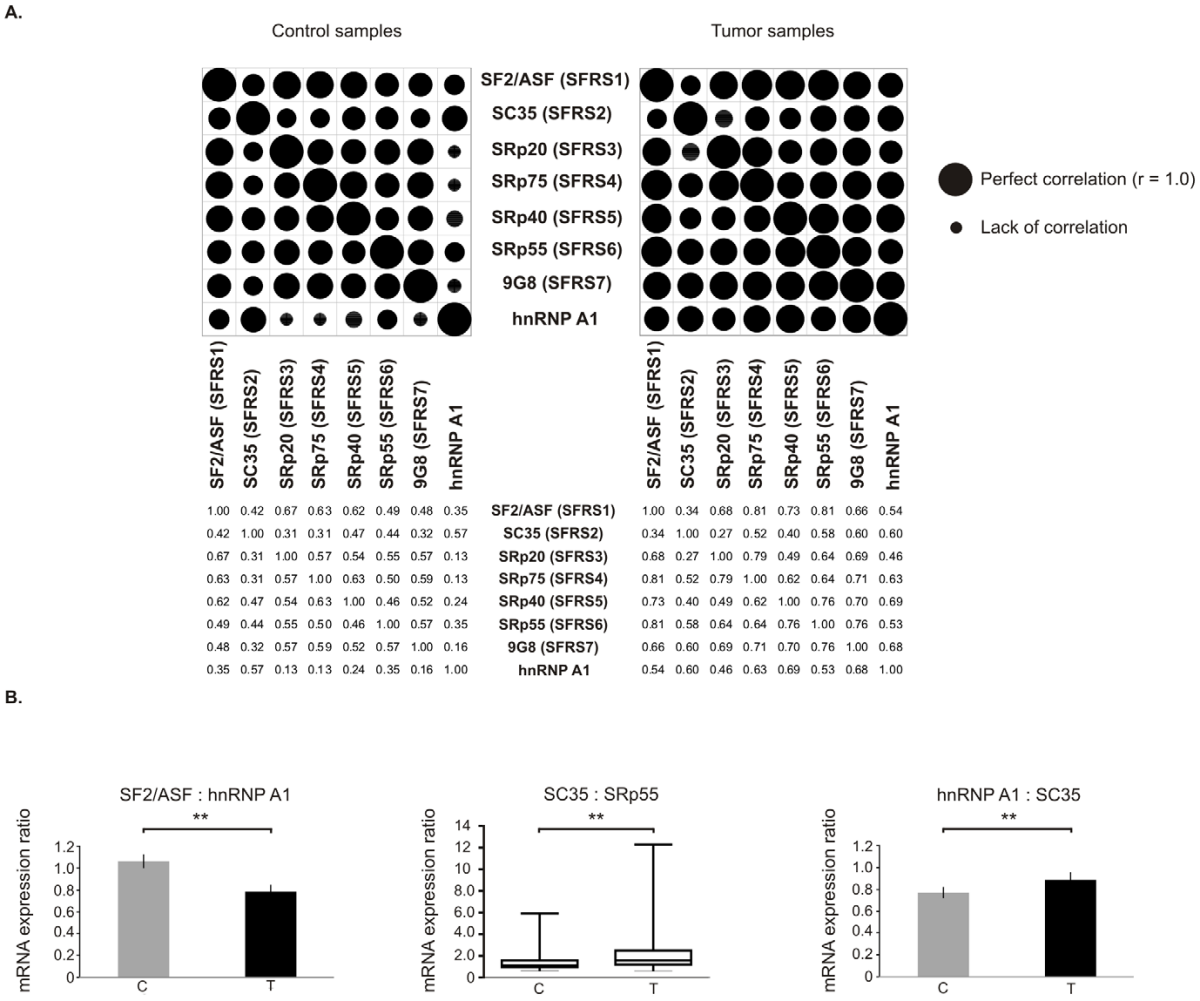
Changes in ratios between splicing factors in tissue samples. **A.** Matrix showing correlation coefficients between mRNA expression of analyzed splicing factors. The plot was generated based on Pearson correlation coefficients between expression values of splicing factors. Patient number 16 was removed from analysis due to deviation from normal distribution. For SC35 (gene: SFRS2) Spearman nonparametric correlation was used as data were not normally distributed in this group. The values of Pearson or Spearman r are given below the dot diagram. Splicing factors' gene names are shown in brackets. P<0.05 was considered statistically significant. **B.** mRNA expression ratios of splicing factors known to act antagonistically. The data are given as mean ± S.E. (for SF2/ASF: hnRNP A1 and hnRNP A1: SC35) or as median values and 95% CI (for SC35: SRp55 as data were not normally distributed in this group). Statistical analysis was performed using paired t test (for for SF2/ASF: hnRNP A1 and hnRNP A1: SC35) or Wilcoxon paired test (for SC35: SRp55). n = 37 for C, n = 37 for T, ** p<0.01.

In order to explore whether changes in expression of splicing factors are correlated, we analyzed ratios between expression levels of splicing factors as well as ratios between specific pairs of splicing factors (Fig. 3). We found that the pattern of correlations differed between control and tumor samples, with general tendency for increased correlation coefficients in tumor samples (Fig. 3A). The strongest changes in correlation were observed for hnRNP A1. For instance, there was no significant correlation between hnRNP A1 and SRp20, hnRNP A1 and SRp75, and hnRNP A1 and 9G8 in control samples, while in tumor samples the expression of these genes correlated significantly. Also, correlation between SF2/ASF and the residual six analyzed splicing factors was stronger in tumor samples.

There are pairs of splicing factors that are known to work antagonistically [13]–[15], [29]. Therefore we analyzed ratios of the following specific splicing factors: SF2/ASF: hnRNP A1, SF2/ASF: SRp20, SF2/ASF: SC35, SRp40: SRp55, SC35: SRp55, and hnRNP A1: SC35. We found that for the three pairs of splicing factors the ratios differed significantly in tumor samples when compared to control samples (Fig. 3B). The SF2/ASF: hnRNP A1 ratio was decreased in tumor samples by about 26% (1.07±0.06 S.E. for C vs 0.78±0.06 S.E. for T; p = 0.0022). The ratio of SC35: SRp55 was increased in tumor samples by about 40% (median 1.12, range 0.60 to 5.91 for C; median 1.57, range 0.59 to 12.29 for T; p = 0.0073). For the ratio hnRNP A1: SC35 there was a small (15.3%) but statistically significant increase in tumors in comparison to control samples (0.77±0.05 S.E. for C vs 0.89±0.064 S.E. for T; p = 0.0197).

### The protein expression of SF2/ASF and hnRNP A1 is disturbed in ccRCC

**Figure 4:**
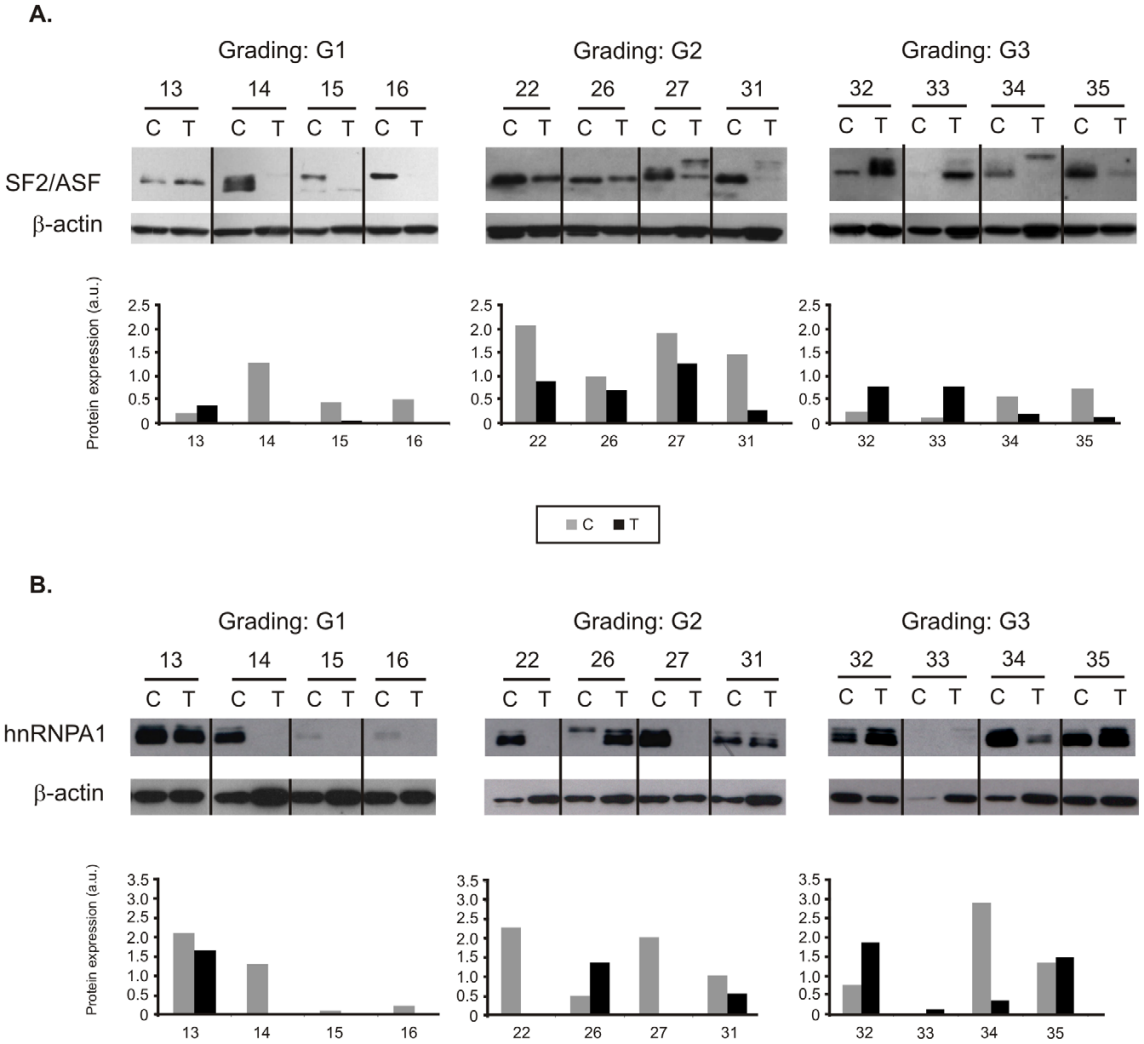
Protein expression of splicing factors in twelve representative pairs of control (C) and tumor (T) samples. Tumor grades of differentiation are shown (G1, G2, G3). Western blots of SF2/ASF (**A**) and hnRNP A1 (**B**) were used for semiquantitative analysis of protein bands after normalization to β-actin. Gray bars represent control samples. Black bars represent tumor samples.

To check whether changes in mRNA level result in concomitant disturbances of protein expression we performed Western blot analysis of two splicing factors, SF2/ASF and hnRNP A1 on twelve representative pairs of control and tumor samples (Fig. 4). Indeed, we found significant differences between protein levels of splicing factors in control and tumor samples. Similarly as in case of mRNA analysis, the changes were variable but did not correlate with mRNA expression. In majority of analyzed paired tissue samples the expression of splicing factor was decreased in samples T in comparison to samples C (SF2/ASF: 9 pairs; hnRNP A1: 8 pairs).

In several samples additional or shifted bands were visible, especially in blots of SF2/ASF (Fig. 4A). Such a picture is characteristic for differently phosphorylated molecules of SF2/ASF protein [30].

### Alternative splicing of genes involved in tumorigenesis and regulated by splicing factors is disturbed in ccRCC

**Figure 5:**
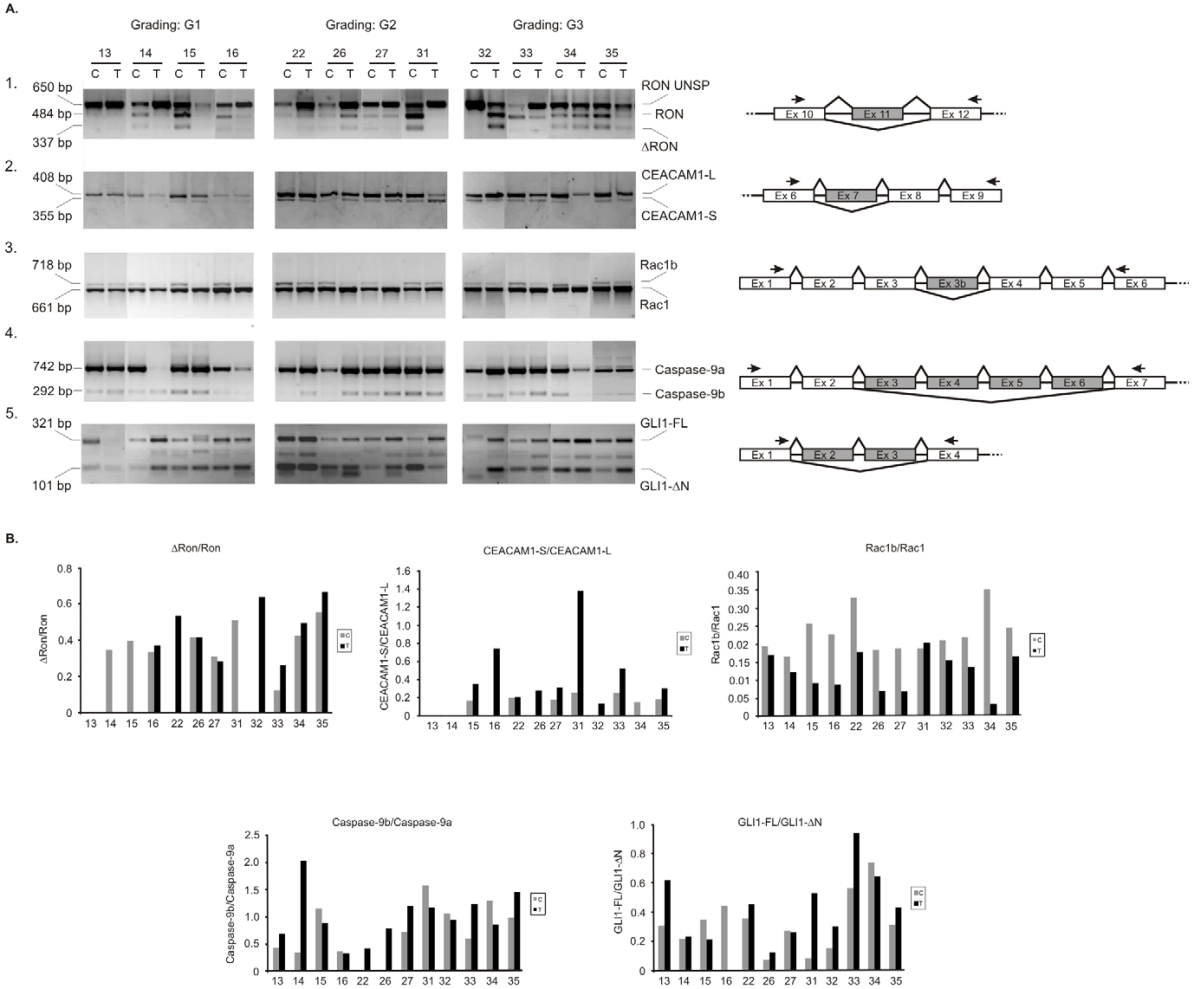
Alternative splicing of genes involved in tumor progression. **A.** PCR analysis of alternative splicing patterns in twelve pairs of control (C) and tumor (T) tissue samples. 1) RON; 2) CEACAM-1; 3) Rac1; 4) Caspase-9; 5) GLI1. Positions of primers used for PCR are shown relative to exons. Alternatively spliced exons are shaded. Gradings of tumor differentiation are shown (G1, G2, G3). **B.** Graph showing expression ratios of splice variants as determined by densitometric analysis of electrophoresed PCR products. Note different axis scales. Gray bars represent control samples. Black bars represent tumor samples.

SF2/ASF regulates alternative splicing of a significant number of genes, including RON proto-oncogene [19], apoptosis regulator Caspase-9 [22], and Rac1, a member of Ras family of proto-oncogenes (regulated by SF2/ASF and SRp20) [14]. To investigate whether alterations in amounts of splicing factors are followed by changes in alternative processing of target genes, we analyzed their splicing patterns (Fig. 5). Furthermore, we analyzed splicing profiles of two additional genes: GLI1 oncogene [25] and CEACAM1 tumor suppressor [23] whose disturbed splicing is known to contribute to tumor progression.

We found that levels of different splicing variants of analyzed genes varied between the majority of the twelve analyzed pairs of control and tumor samples in which protein level of SF2/ASF and hnRNP A1 was analyzed (Fig. 5B). Expression of RON proto-oncogene splice variants Ron and ΔRon differed between samples of specific grades of differentiation. In all G3 tumor samples the ratio ΔRon/Ron was higher than in paired control samples. In samples G1 tumor-specific ratio ΔRon/Ron was higher or similar in comparison to control samples. In samples taken from patient number 13 both isoforms were absent in tumor and control samples. In tumor samples classified as G2 the ratio ΔRon/Ron was variable: in two tumor samples it was similar as in paired controls, in one sample it was higher than in paired control and in one sample it was lower than in paired control. Splicing patterns of CEACAM1 did not depend on the differentiation grade of tumor sample. In seven sample pairs the ratio CEACAM1-S/CEACAM1-L was higher in tumors than in control samples. In two sample pairs CEACAM1-S was not detected. Splicing pattern of Rac1 was similar in majority of analyzed samples. The ratio Rac1b/Rac1 was higher in control samples than in paired tumors in eleven of analyzed tissue pairs. Seven of analyzed sample pairs revealed higher ratio of Caspase-9b/Caspase-9a in tumors than in control samples independently of differentiation grades. Splicing pattern of GLI1 was the most variable. The ratio of GLI1-FL/GLI1-ΔN in seven tumor samples was higher than in paired controls. In two pairs of samples there were no differences between ratios of analyzed splice variants; however additional bands were visible, suggesting presence of not identified splice variants.

**Figure 6:**
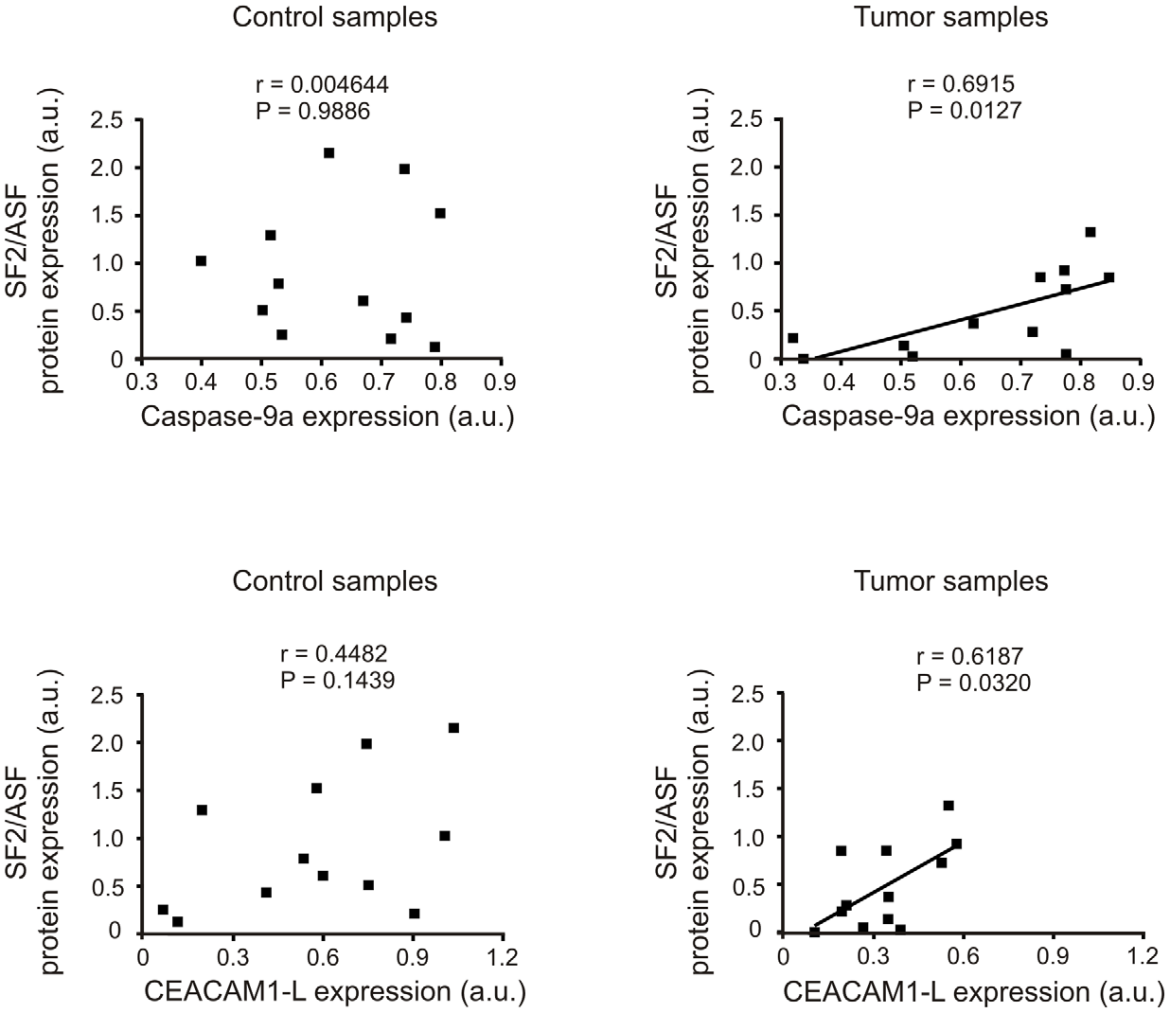
Correlation between protein expression of SF2/ASF and Caspase-9a (A) and CEACAM1-L (B). Pearson correlation analysis was performed on data from twelve pairs of control and tumor tissue samples. P<0.05 was considered statistically significant.

In order to find possible connection between changes in expression of SF2/ASF and splicing of SF2/ASF-regulated genes we performed Pearson correlation analysis between the expression of splicing factors and the target exon splicing (Fig. 6). Positive correlation (r = 0.6915, p = 0.0127) between Caspase-9a and SF2/ASF protein was observed in tumor but not in control samples (r = 0.004644, p = 0.9886). We also found a positive correlation (r = 0.6187, p = 0.0320) between CEACAM1-L and SF2/ASF protein in tumors but not in control samples (r = 0.4482, p = 0.1439). We did not find any correlation between the expression of SF2/ASF (or hnRNP A1) proteins and the splicing variants of Ron, Rac1 or GLI-1 (data not shown).

**Figure 7:**
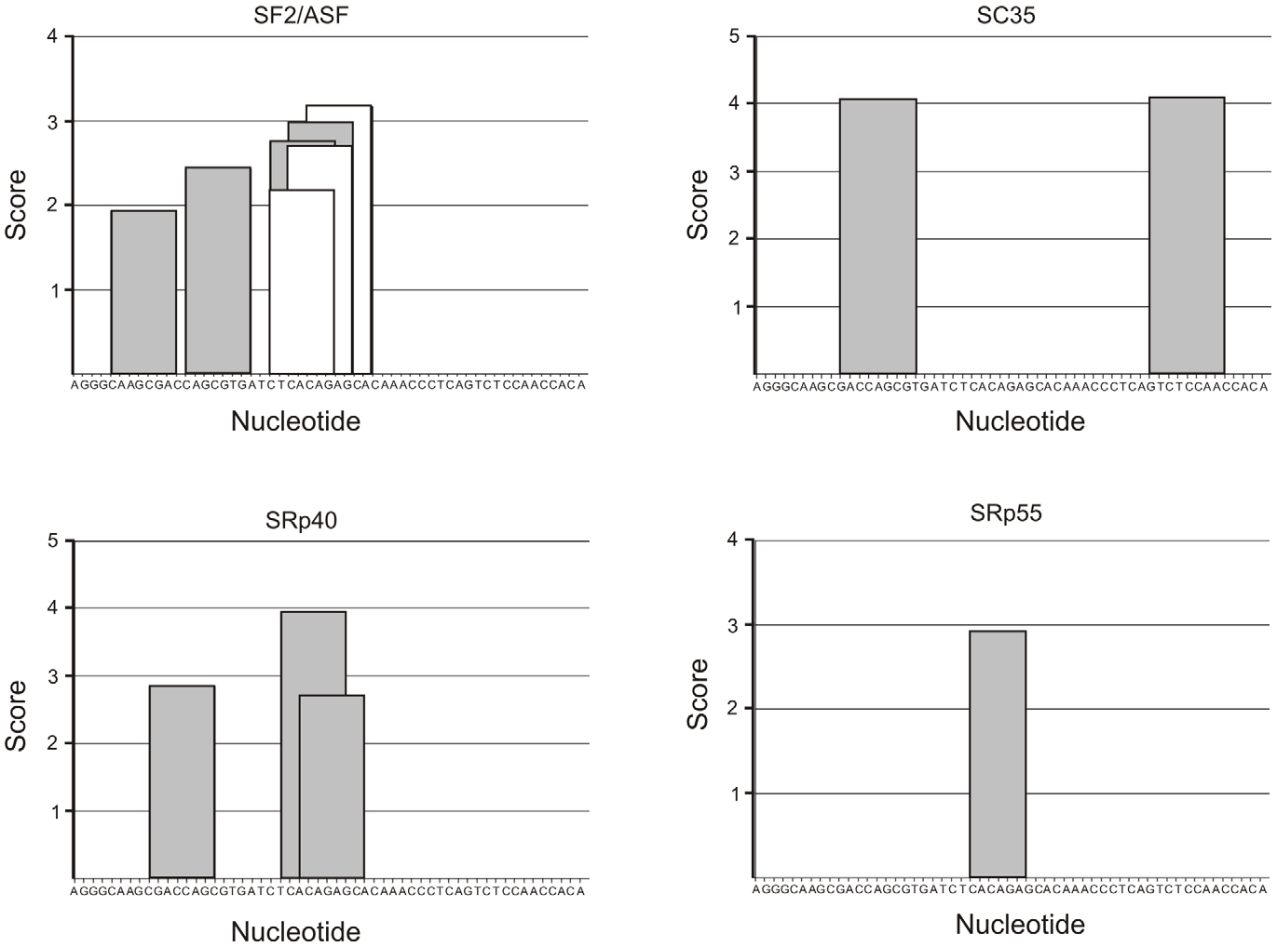
Analysis of CEACAM1 exon 7 sequence in a search for potential binding motifs of splicing factors. Prediction of motifs was performed with ESE Finder software [26] using matrices for prediction of sequences required for binding of splicing factors. For the prediction of SF2/ASF binding sites, two matrices were used: “SF2/ASF/IgM-BRCA1“ (white bars) and “SF2/ASF“ (gray bars). These two matrices were derived in different context (different minigenes and size of random sequence libraries in SELEX [27]). Only high-score motifs above thresholds for SF2/ASF (1.956), SF2/ASF/IgM-BRCA1 (1.867), SC35 (2.383), SRP40 (2.670), and SRp55 (2.676) are shown. The nucleotide sequence of CEACAM1 exon 7 is given on x-axis.

While it is known that SF2/ASF regulates splicing of Caspase-9 [22], there are no data showing that CEACAM1-L is a target of SF2/ASF or of any other splicing factors. However, it was found that exon 7 of CEACAM1 contains cis-acting splicing regulatory elements [23]. Therefore we analyzed the sequence of CEACAM1 exon 7 using matrices for prediction sequences required for binding of splicing factors SF2/ASF, SC35, SRp40, AND SRp55 (Fig. 7). This analysis revealed several high-score motifs for SF2/ASF, two motifs for SC35, three motifs for SRp40, and one motif for SRP55.

## Discussion

In this paper we show that mRNA expression of eight splicing factors is disturbed in ccRCC. The protein expression of two splicing factors, known to act antagonistically, SF2/ASF and hnRNP A1 is also disturbed. These aberrations are accompanied by impaired alternative splicing of SF2/ASF dependent genes, RON, Caspase-9, and Rac1. Tumor-specific disturbances of alternative splicing were also found for GLI1 oncogene and CEACAM1 tumor suppressor.

Changes in pre-mRNA splicing are a common phenomenon in human malignancies. According to our findings, improperties in expression of splicing factors occur in at least half (50–60%) of analyzed samples, although the disturbances are diverse and result from both up- and downregulation of splicing factors (Fig. 1 and 2). The direction of changes is not specific for tumor grades of differentiation since both up- and downregulation were observed in all gradings. It is notable that although most of the publications refer to tumor-specific increase of splicing factors expression, the percentage of samples in which the disturbed expression occurs is rarely shown. Karni et al. [18] reported that among 50 analyzed ccRCC samples more than 2-fold overexpression of SF2/ASF mRNA was observed in less than 5% of samples. According to our analysis the percentage of samples with more than 2-fold overexpression was a little higher (8%) but this difference may result from relatively small number of analyzed samples.

Interestingly, we found that patterns of mRNA and protein expression of splicing factors differed between individual patients and also between control and tumor samples of a given patient. This is in agreement with our previous study showing tumor-specific and patient-specific splicing patterns of type 1 iodothyronine deiodinase [4] and with other studies, showing that ccRCC is characterized by molecular heterogeneity and can be separated into gene expression subgroups [31]–[33]. In the study of Zhao et al. [33] those expression subgroups correlated with survival after surgery but, similarly as in our study, did not correlate with tumor grades. Unfortunately, in case of our study the patients were not followed up thus it is not possible to analyze any correlations between expression profiles and patients survival. Klein et al. [34] analyzed single residual tumor cells from several types of tumors (but not of kidney origin) and found high heterogeneity of gene expression between particular cancer cells. Those cells were heterogeneous irrespective of whether they resided within the same compartment or within different homing sites. Patient specific variation in gene expression was also reported for breast and prostate cancer [35], [36].

This gene expression variability is an interesting issue, and at least two possible origins should be considered: that the variations reflect the tumor specificity or that they reflect individual patient features that result in a specific picture of gene expression in tumor. The first situation was described for liver cancer in which separate tumors from a single patient showed dramatic differences in gene expression patterns [37]. On the other hand, Perou et al. [35] reported that gene expression patterns of different breast tumor samples taken from one patient were more similar to each other than to samples taken from other patients. It is not clear which situation concerns our study as we analyzed single samples per patient. As we have recently shown, however, ccRCC tumors may also reveal more homogenous gene expression patterns [6]. In that study in which we used partially the same material as in this paper, we found highly consistent ccRCC tumor specific disturbances of thyroid hormone pathway: decrease of thyroid hormone receptor β1 (TRβ1) mRNA and protein, loss of type 1 iodothyonine deiodinase protein and lowered level of thyroid hormone, triiodothyronine (T3). Interestingly, TRβ1 protein decrease was observed in all twelve pairs of tumor and control samples that were subsequently used for our study. These observations are in agreement with findings of Takahashi et al., [31] who identified groups of genes that were similarly or differentially expressed in ccRCC. Thus, the consistent changes in thyroid hormone pathway may possibly represent the cell from which the tumor originated, while different gene expression patterns of splicing factors may reflect later events in tumor growth, including mutations as well as epigenetic and chromosomal alterations.

We observed increase of Pearson coefficients between mRNA expression of splicing factors in tumors (Fig. 3). These positive correlations were observed in tumors collected from different patients and confirmed that the observed changes in expression result from cancer specific disturbances in tumor tissue and not from individual differences between patients. Coordinated changes in expression of splicing factors may suggest the existence of not yet identified factor that affects the expression of the whole group of genes involved in splicing. Our observations are consistent with findings of Warrenfeltz et al. [38] who found that functionally related genes displayed correlated changes of expression in ovarian cancer. The coordinated changes of gene expression may result from several causes. Genes that are located in similar regions of chromatin may be concomitantly influenced by changes in chromatin structure or by changes in chromosome regions. The majority of genes analyzed in our study is located on different chromosomes (apart from SFRS1 and SFRS2 that are located on chromosome 17), thus this effect is rather not responsible for the observed changes. Another reason could be the changed activity of a transcription factor that regulates expression of analyzed splicing factors. Such co-regulation of several splicing factors by one factor is possible, as shown by Mole et al. [39], who discovered that expression of SF2/ASF, SC35 and SRp75 is upregulated by human papillomavirus HPV16 infection, possibly via activation of transcription, controlled by the viral transcription factor E2. There are known transcription factors whose expression is changed in ccRCC [6], [40], however the question whether they are also regulators of splicing factors remains for further investigation.

The relative amounts of SR- and non-SR splicing factors, such as hnRNP A1, may determine patterns of alternative splicing of many genes. We found that relative amounts of pairs of splicing factors that are known to act antagonistically statistically significant differed in tumors in comparison to paired control samples. This was especially visible for antagonistic pair SF2/ASF and hnRNP A1 whose ratio is known to influence 5′ splice site selection [13]. When the level of hnRNP A1 is higher than of SF2/ASF, distal 5′ splice site is preferred. In our work the ratio SF2/ASF: hnRNP A1 was significantly lowered in ccRCC samples in comparison to controls (Fig. 3B). This result is in agreement with our previous analysis [4] in which the predominant variants of type 1 iodothyronine deiodinase (DIO1) found in ccRCC were those resulting from distal 5′ splice site usage.

Apart from disturbed expression, several other factors may also contribute to improperties in functionality of splicing factors. One of them could be disturbed protein phosphorylation, the modification which determines activity of splicing factors [41]. Of note, Western blot analysis of SF2/ASF and hnRNP A1 revealed additional or shifted bands that are characteristic for differently phosphorylated proteins [30]. One of kinases pathway that regulate phosphorylation of splicing factors is PI3K/Akt [42]. Interestingly, this pathway is disturbed in ccRCC [43] and thus may possibly contribute to impaired activity of splicing factors. To confirm, however, that phosphorylation of splicing factors is disturbed in ccRCC, further analysis is needed. Another posttranslational modification affecting the activity of splicing factors is poly(ADP-ribosyl)ation [44]–[46]. There are no direct data showing changes in poly(ADP-ribose) polymerase activity in ccRCC; it was shown, however, that nephrocarcinogens induce poly(ADP-ribosyl)ation of renal proteins [47]. There are also other modifications that may affect the activity of splicing factors such as sumoylation [48], and arginine methylation [49], [50]; however further studies are needed to reveal whether they play any role in ccRCC pathology.

Disturbances of alternative splicing directly contribute to tumorigenesis due to synthesis of alternative products that exert tumor growth promoting activities [51]. Splicing factors themselves may also act as proto-oncogenes, as it was shown for SF2/ASF [18]. The complex changes of eight splicing factors in ccRCC samples found in our work may thus result in alterations of splicing reactions. Indeed, we show here that alternative splicing of five genes is disturbed in analyzed tumor tissues. Importantly, these genes are known to influence the process of tumorigenesis. We found a positive correlation between Caspase-9a and SF2/ASF protein in tumor but not in control samples. Similar positive correlation was found between CEACAM1-L and SF2/ASF protein (Fig. 6.). Positive correlation between expression of SF2/ASF and Caspase-9a is in agreement with the observation that SF2/ASF enhances the expression of Caspase-9a isoform [22]. Lack of correlation between Caspase-9a/9b ratio suggests that additional factors may influence the splicing of Caspase-9 as for instance selective degradation of splice variant 9b or regulatory effect of other proteins. It was shown that splicing of Caspase-9 is affected by E2F1 and SC35 [52]. Interestingly, as we showed, the expression of E2F1 is disturbed in ccRCC [53]. The observation that the expression of SF2/ASF and Caspase-9a correlates positively in tumorous but not in control samples may possibly suggest that while in non-cancerous kidney tissues the splicing of Caspase-9 is affected by multiple different factors (for instance, by E2F1 and SC35), in cancer tissues SF2/ASF may play the main role.

The pathway of programmed cell death is disturbed in ccRCC due to loss of apoptosis inducers [54] or upregulation of apoptosis inhibitors [55]. Our results showing changed ratios of proapoptotic [9a] and antiapoptotic [9b] Caspase-9 splice variants in tumor tissue samples resemble those found for survivin, a member of the inhibitor of apoptosis protein (IAP) family [9]. Mahotka et al. [9] analyzed survivin splice variants that showed different antiapoptotic properties and found that the mRNA ratio between survivin-2B (the isoform lacking antiapoptotic activity) and survivin is decreased in late tumor stages of ccRCC. Disturbances of apoptotic pathway are also confirmed in this study, showing imbalanced ratios of GLI1 variants. GLI1 is a transcription factor of oncogenic potential [56] that increases expression of a number of antiapoptotic factors. Moreover, it was shown that GLI1 mRNA expression is upregulated in ccRCC [57]. Together these results suggest that ccRCC specific disturbances of apoptosis may result not only from general changes of levels of apoptotic regulators but also from perturbations of alternative splicing.

CEACAM1 is an adhesion molecule of particularly broad effects on cancerous growth and invasion [58]. It affects cell adhesion, apoptosis, morphogenesis, cell proliferation, invasion, cell migration, angiogenesis, lymphangiogenesis and cytotoxicity. The activity of CEACAM1 is regulated by alternative splicing that generates two types of cytoplasmic domain: short (present in CEACAM1-S isoform) and long (present in CEACAM1-L) one. Both CEACAM1-L and CEACAM1-S were shown to inhibit tumor growth when transfected and expressed in different types of cancer [59]–[61]. It was suggested that the ratio of S: L isoforms may define suppressive properties of particular isoforms [23] and that CEACAM1-L activity may be inhibited by CEACAM1-S [62]. Our results showing increased S:L ratio in tumor cells are consistent with antagonistic properties of the two CEACAM1 isoforms. Interestingly, Kammerer et al. [63] found that CEACAM1 protein is completely lost in ccRCC; however, this group did not analyze CEACAM1 mRNA level or splicing patterns in cancer tissues. There are no data showing that CEACAM1-L is a target of SF2/ASF or any other splicing factor analyzed in our study. However, Gaur et al. [23] identified cis-acting splicing regulatory elements located in exon 7 of CEACAM1. Analysis of CEACAM1 exon 7 revealed several high-score motifs for SF2/ASF, SC35, SRp40, and SRP55 (Fig. 7). Although a score above threshold does not necessarily mean that the sequence functions as an exonic splicing enhancer, these results together with positive correlation between the level of CEACAM1-L and SF2/ASF and the presence of regulatory cis acting elements in CEACAM1 exon 7 suggest that SF2/ASF may play a regulatory role in CEACAM1 splicing. This issue needs further confirmation by experimental data showing direct binding between CEACAM1 exon 7 and SF2/ASF.

We did not find any correlation between SF2/ASF (or hnRNP A1) and the splicing pattern of Ron, Rac1 or GLI-1 which may possibly suggest that other factors may play a role in regulation of these genes' splicing. RON tyrosine kinase is a proto-oncogene that controls invasive growth of tumors [64]. Alternatively spliced isoforms of RON found in cancers promote changes in tumor cell morphology, stimulate proliferation and abrogate cell-cell adhesion [19], [65]. RON dependent cell motility is regulated by SF2/ASF that controls alternative splicing of this protooncogene [19]. It was shown that ccRCC tumors classified as G3 and G2 differentiation grades have higher metastasis rates compared to that of grade G1 [66]. As ΔRON is a constitutively active isoform that triggers invasive tumor growth and whose accumulation is observed in colon and breast cancers [19], [67], our results showing higher tumor-specific ΔRON/RON ratio in all tumor samples classified as G3 grade are in line with those observations.

Rac1 is a member of Rho-like GTPases that affect changes in actin cytoskeleton and regulate cell adhesion, migration and invasion [68]. Alternative splicing regulates cancer-related properties of Rac1, leading to synthesis of constitutively active Rac1b, whose expression is upregulated in cancers [24]. Moreover, Rac1b expression in NIH-3T3 cells causes growth transformation [69] and determines survival of colon cancer cells [70]. Our results showing that ratio of Rac1b: Rac1 is higher in controls than in ccRCC samples suggest that this isoform rather does not play tumor promoting role in ccRCC. This is in line with previous findings of Engers et al. [71] who showed that forced expression of Rac1 in ccRCC cells leads to inhibition of cell migration and invasion. This group, however, did not look at the splicing profile of Rac1, therefore the exact role of Rac1b in ccRCC remains for further investigations.

In conclusion, we show that changes in expression of splicing factors belonging to SR proteins family and a non-SR protein, hnRNP A1, may possibly lead to disturbances of alternative splicing in ccRCC. These disturbances may potentially be directly connected with tumor progression as they result in impaired alternative splicing of genes involved in apoptosis, and cell adhesion. We suggest that changes in correlation between expression levels of splicing factors may potentially serve as markers of carcinogenesis in ccRCC and that splicing factors may possibly constitute therapeutic targets in patients with disturbed expression of specific splicing regulators. This last possibility was already successfully tested on cancer cell lines by Patry et al. [72].

## Supporting Information

Table S1 Primers used for PCR analysis of splicing profiles. Sequences of primers were taken from the previously published reports (referenced).
(0.03 MB DOC)

Table S2 Primers used for real-time PCR analysis.
(0.03 MB DOC)

Figure S1 Expression of 18sRNA and ACTB housekeeping genes in paired control-tumor samples. Upper of each panel shows crossing points (threshold cycle) obtained while measuring the gene expression in each sample, whereas bottom of each panel shows crossing points plotted against the log concentration to obtain a standard curve. Gray bars represent control samples, black bars represent tumor samples.
(0.51 MB DOC)

Figure S2 Patient-specific profiles of expression of splicing factors. The plots show mRNA expression of each gene, normalized to 18sRNA, measured in triplicate. Gray bars represent control samples, black bars represent tumor samples Results are shown as mean +/− S.E. Statistical analysis was performed using t-test.
(1.04 MB DOC)
