# Supplementary material for: Disturbed Expression of Splicing Factors in Renal Cancer Affects Alternative Splicing of Apoptosis Regulators, Oncogenes, and Tumor Suppressors
Source: PLoS One. 2010 Oct 27;5(10):e13690. doi: 10.1371/journal.pone.0013690 (PMC2972751; doi:10.1371/journal.pone.0013690)
Supplement: Table S1 — Primers used for PCR analysis of splicing profiles. Sequences of primers were taken from the previously published reports (referenced). (0.03 MB DOC) [file pone.0013690.s001.doc]

**Table S1. Primers used for PCR analysis of splicing profiles.** Sequences of primers were taken from the previously published reports (referenced).

| **Target transcript** | **Primers: forward (F) and reverse (R)** | **Reference** |
| --- | --- | --- |
| RON | F: CCTGAATATGTGGTCCGAGACCCCCAG  R: CTAGCTGCTTCCTCCGCCACCAGTA | [19] |
| CEACAM1 | F: GGTTGCTCTGATAGCAGTAG  R: AGCCTGGAGATGCCTATTAG | [23] |
| Rac1 | F: TTCCTATCTCAGCGCCCTGC  R: GGACAGGACCAAGAACGAGGG | [24] |
| Caspase-9 | F: GCTCTTCCTTTGTTCATCTCC  R: CATCTGGCTCGGGGTTACTGC | [22] |
| GLI1 | F: GAGCCCAGCGCCCAGACAGA  R: GGCATCCGACAGAGGTGAGATGGAC | [25] |
