# Supplementary material for: Disturbed Expression of Splicing Factors in Renal Cancer Affects Alternative Splicing of Apoptosis Regulators, Oncogenes, and Tumor Suppressors
Source: PLoS One. 2010 Oct 27;5(10):e13690. doi: 10.1371/journal.pone.0013690 (PMC2972751; doi:10.1371/journal.pone.0013690)
Supplement: Table S2 — Primers used for real-time PCR analysis. (0.03 MB DOC) [file pone.0013690.s002.doc]

**Table S**2. Primers used for real-time PCR analysis.

| **Target Gene** | **Primers: forward (F) and reverse (R)** |
| --- | --- |
| SFRS1 | F: TTAGATCTCATGAGGGAGAAA  R: GAGAATAGCGTGGTGATCCT |
| SFRS2 | F: ACAAGCGCGACGCTGAGG  R: CTGCGAGACCTGGAACGG |
| SFRS3 | F: TTTTGGCTACTATGGACC  R: AGTTCCACTCTTACACGG |
| SFRS4 | F: ACGGGAAGATCCTGGAGG  R: ACTCGCTCACCACAAAGG |
| SFRS5 | F: CGTTTGCGGATGCACACC  R: TAGAGGAACTTCTGGTCC |
| SFRS6 | F: TGTTCGTACAGAATACAGG  R: TTTATTTCTGTGCCATCC |
| SFRS7 | F: TCACTCAGAAGATCTAGG  R: TCAGTCCATTCTTTCAGG |
| hnRNPA1 | F: TGGATTTGGTAATGATGGAAGC  R: TCTCTGGCTCTCCTCTCCTG |
| 18SRNA | F: GTAACCCGTTGAACCCCATT  R: CCATCCAATCGGTAGTAGCG |
