# Supplementary material for: Disturbed Expression of Splicing Factors in Renal Cancer Affects Alternative Splicing of Apoptosis Regulators, Oncogenes, and Tumor Suppressors
Source: PLoS One. 2010 Oct 27;5(10):e13690. doi: 10.1371/journal.pone.0013690 (PMC2972751; doi:10.1371/journal.pone.0013690)

**Fig. S1. Expression of 18sRNA and ACTB housekeeping genes in paired control-tumor samples.** Upper of each panel shows crossing points (threshold cycle) obtained while measuring the gene expression in each sample, whereas bottom of each panel shows crossing points plotted against the log concentration to obtain a standard curve. Gray bars represent control samples, black bars represent tumor samples.


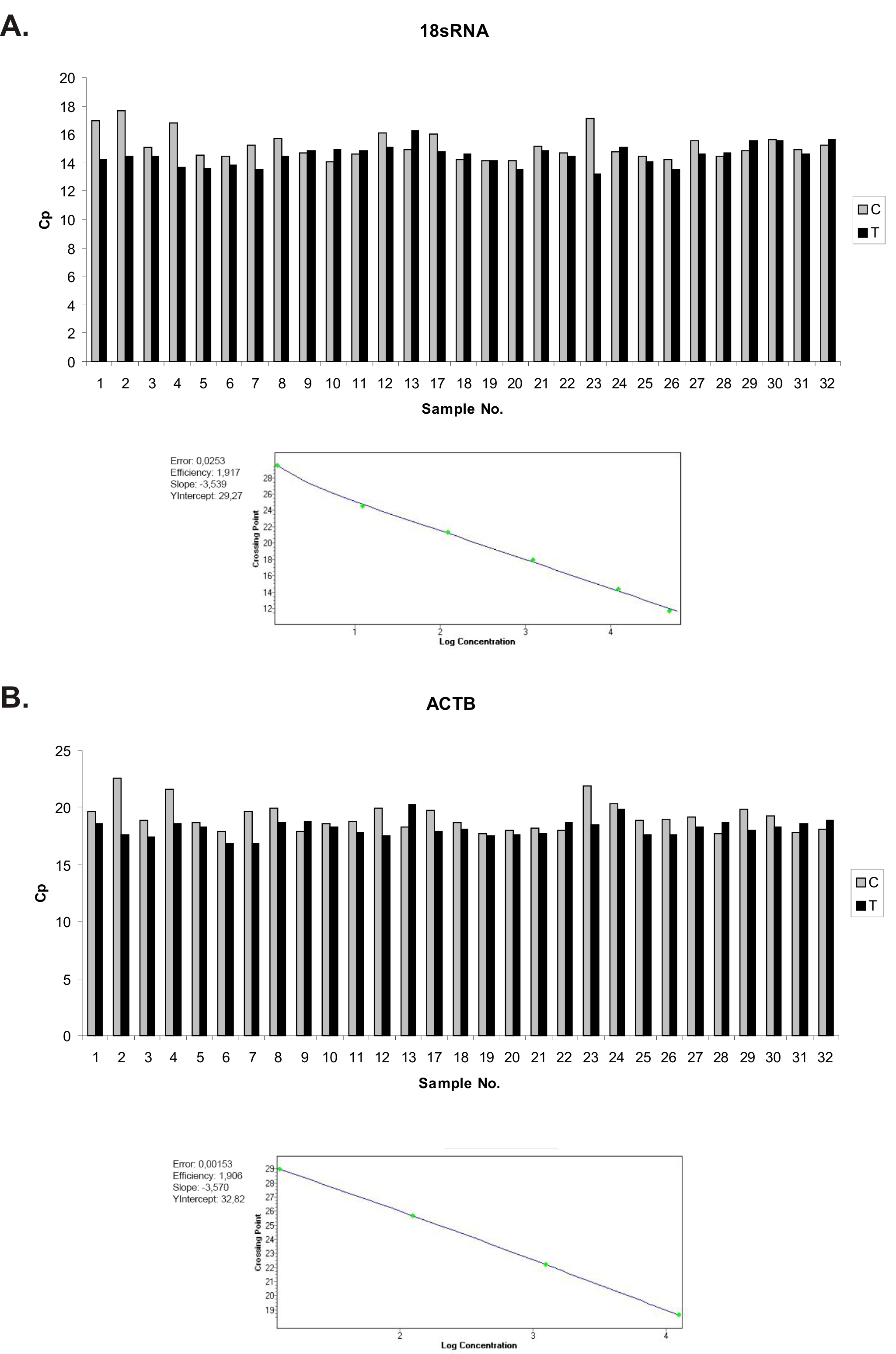

Supplement: Figure S1 — Expression of 18sRNA and ACTB housekeeping genes in paired control-tumor samples. Upper of each panel shows crossing points (threshold cycle) obtained while measuring the gene expression in each sample, whereas bottom of each panel shows crossing points plotted against the log concentration to obtain a standard curve. Gray bars represent control samples, black bars represent tumor samples. (0.51 MB DOC) [file pone.0013690.s003.doc]
