# Supplementary material for: Disturbed Expression of Splicing Factors in Renal Cancer Affects Alternative Splicing of Apoptosis Regulators, Oncogenes, and Tumor Suppressors
Source: PLoS One. 2010 Oct 27;5(10):e13690. doi: 10.1371/journal.pone.0013690 (PMC2972751; doi:10.1371/journal.pone.0013690)

**Fig. S2. Patient-specific profiles of expression of splicing factors.** The plots show mRNA expression of each gene, normalized to 18sRNA, measured in triplicate. Gray bars represent control samples, black bars represent tumor samples Results are shown as mean ± S.E. Statistical analysis was performed using t-test.


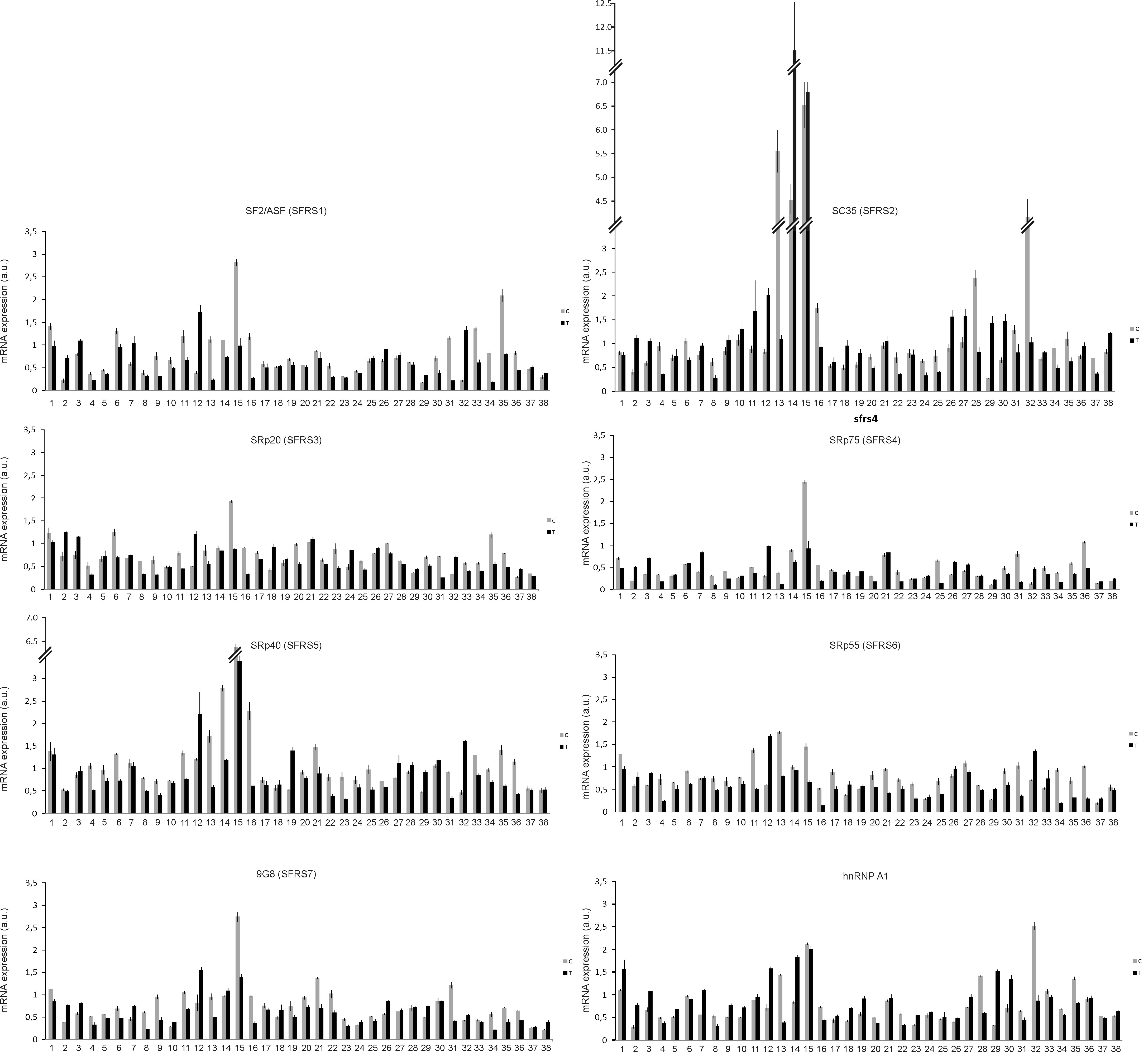

Supplement: Figure S2 — Patient-specific profiles of expression of splicing factors. The plots show mRNA expression of each gene, normalized to 18sRNA, measured in triplicate. Gray bars represent control samples, black bars represent tumor samples Results are shown as mean +/− S.E. Statistical analysis was performed using t-test. (1.04 MB DOC) [file pone.0013690.s004.doc]
